# Perceptions of Health Effects of Electronic Cigarettes in Young Adults: Emergency Department Patients vs. Medical Students

**DOI:** 10.5811/westjem.50709

**Published:** 2026-04-08

**Authors:** Hope Smelser, Cameron Heying, Lindsay Maguire

**Affiliations:** * University of Kansas School of Medicine, Wichita, Kansas; † The University of Kansas Health System, Kansas City, Kansas; ‡ University of Kansas School of Medicine, Kansas City, Kansas

## Abstract

**Introduction:**

As electronic cigarette (e-cigarette) use becomes more prevalent, understanding how populations perceive the harms associated with use is vital for tailoring public health interventions. Our aims in this study were to explore the perceptions of health risk associated with e-cigarettes among young patients in the emergency department (ED) who consume e-cigarettes as well as similarly aged medical students regardless of e-cigarette use and to determine medical students’ perception of their curriculum to prepare them for future counseling of patients on e-cigarette use.

**Methods:**

A cross-sectional survey was completed by 276 participants: 90 ED patients 18–35 years of age who had ever used e-cigarettes (4.2% response rate) and 187 medical students from a U.S. allopathic medical school (17.7% response rate). Our primary outcomes were perceptions of health risks associated with e-cigarette use and medical student perceptions of the medical school curriculum. The secondary outcome was perceptions of e-cigarettes compared to tobacco cigarettes and perceptions of medical students’ readiness to counsel patients on e-cigarette use. Bivariate analyses using chi-square tests assessed differences between groups.

**Results:**

We received 90 completed surveys from ED patients, and 187 from medical students. The majority of ED patients reported believing that e-cigarette use can lead to lung injury (77.8%), heart disease (30%), and cancer (82.2%). Medical students were more likely than ED patients to associate e-cigarette use with harm (lung injury, 94.7% vs 77.8%, *P* < .001; heart disease, 84.0% vs 70.0%, *P* = .007; and cancer 90.9% vs 82.2%, *P* = .037). A modest proportion of ED respondents stated that e-cigarette use did not carry risk of lung injury (22.2%), heart disease (30%), and cancer (17.2%). Most medical students (61.0%) believed that their medical school curriculum did not prepare them for future conversations with patients about e-cigarettes, and over half of the students (54.0%) expressed low confidence in counseling patients.

**Conclusion:**

In our population, a significant proportion (20–30%) of ED patients did not perceive risk with e-cigarette use, suggesting room for education and intervention in this population. Medical education is likely associated with increased awareness of risk of e-cigarette use. Medical students generally did not feel prepared for the growing need to counsel patients on e-cigarette use, suggesting medical curricula could be adapted to meet this need.

## INTRODUCTION

Electronic cigarette (e-cigarette) use among all United States (US) adults is becoming more prevalent, ranging from 4.5–5.4% over 2016–2021.^1^,^2^ Among young adults (18–24 years of age), use has increased from 9.2% to 15.0% over the same period. Those from rural areas and those who have completed a General Education Development test or lower level of education have been reported to use e-cigarettes at higher rates than their peers.^2^ It has been suggested that higher educational attainment is associated with decreased rates of e-cigarette use.^3^ These factors are often inter-related, as a high percentage of people from rural areas (42%) have a high school education or less.^4^

While data regarding the long-term health effects of e-cigarettes are scarce due to the relative recency of their popularity, e-cigarettes have been shown to pose unique harms with short-term use. E-cigarette-associated lung injuries are known to occur, including lung infections and popcorn lung.^5^ As of February 2020, the U.S. Centers for Disease Control and Prevention (CDC) reported that 2,807 individuals were hospitalized secondary to e-cigarette-associated lung injuries.^6^ Of those hospitalized, accompanying demographic data from the CDC reveals that 15% were < 18 years of age, 37% were 18–24, and 24% were 25–34 years of age. Additionally, e-cigarettes have been implicated in cases of lung cancer.^7^ Use of e-cigarettes has also been associated with increased odds of acute respiratory distress syndrome, coagulopathies, and myocardial infarction.^8^,^9^ Analysis of National Health Interview Survey data indicated that e-cigarette users were 79% more likely to experience a myocardial infarction as compared to non-users.^10^

While the scientific community accumulates data regarding the health effects of e-cigarette use, scientific literature assessing adults’ perceptions of these health effects is limited. Understanding these perceptions is crucial, as perceptions of health effects can be linked to behavior change and influence the development of community outreach and education efforts, including those targeting patients presenting to an emergency department (ED). Brief interventions in the ED have shown modest to moderate success across substances in inciting behavior change. A randomized trial by Bernstein et al found that a multicomponent ED-based intervention significantly improved quit rates among smokers with co-occurring substance use disorders.^11^ Another trial demonstrated that ED-based motivational interviewing reduced marijuana use among young adults 18–25 years of age and identified social media follow-up as a potential target for intervention.^12^ Similar brief interventions could be implemented in the ED to address e-cigarette use.

Perceptions of adverse health effects caused by e-cigarettes have been correlated with an individual’s likelihood to use e-cigarettes, but little research has been done to show what perceptions are commonly held by users. We had two major aims in this study. First, we sought to explore perceptions of the health effects of e-cigarette use held by young adults who presented to an ED, as well as similarly aged medical students. Data gathered from this investigation were intended to drive development of brief intervention materials for use in the ED. We aimed to recruit patients in the same age bracket as medical students to identify whether medical education may drive differing perceptions of e-cigarette use. Second, we aimed to determine medical students’ attitudes toward the quality of their undergraduate medical education curriculum and their comfort in counseling patients on e-cigarette use, to identify potential areas for improvement in medical education.

**Population Health Research Capsule**

What do we already know about this issue?

*Little is known about the perceived health risks of e-cigarette use, but data supports that those who are aware of health risks are less likely to partake*.

What was the research question?

*Do medical students and emergency department patients who use e-cigarettes perceive health effects of e-cigarettes similarly?*

What was the major finding of the study?

*Compared to patients, medical students reported awareness of higher risk of lung injury (95% vs 78%, P < .001), heart disease (84% vs 70%, P = .007), and cancer (91% vs 82%, P = .04)*.

How does this improve population health?

*Targeted education and improvements to medical school curricula could lead to enhanced counseling of e-cigarette users and increased rates of cessation*.

## METHODS

### Participants

Two populations were recruited to participate in this study. First, a convenience sample of patients 18–35 years of age who visited the ED at a major metropolitan academic hospital were recruited from April 5, 2024–December 31, 2024. Patients who reported present or past use of e-cigarettes and fluency in written English were included. No other specific exclusion criteria were used. For the student cohort, we recruited medical students from all four years of a single U.S. allopathic state medical school with three campuses across the state, regardless of e-cigarette use history.

### Instruments

Study participants were surveyed about their basic demographics (age, race, education, sex, ethnicity); their history with and frequency of e-cigarette and cigarette use; and their knowledge and perceptions of and concern about e-cigarette-related lung injury and cardiovascular health implications. Additionally, the medical student sample was asked to provide their level of medical education, their confidence in counseling patients on the use and harms of nicotine products, and their perception of the adequacy of their medical school curricula in including e-cigarette-related topics.

### Procedures

This study was approved by the local institutional review board. For the ED population, potential participants were identified by ED staff trained on study procedures after patients were brought to an examination room and given a flyer with a link to the survey materials. The survey was available between April–November 2024. For the student sample, a flyer describing the study with a link to the survey was sent to the email addresses of University of Kansas Medical Center medical students, requesting participation. The study flyer was distributed by email to all medical students once in the spring semester of the 2023–2024 academic year and once during the fall semester of the 2024–2025 academic year. Only one response per participant was permitted. Participants were eligible for a drawing to receive one of 10 $50 Visa gift cards through a raffle. All survey responses were gathered using Research Electronic Data Capture (REDCap) for data management (CTSA Award # UL1TR002366), hosted at University of Kansas Medical Center. 13,14

### Statistical Analysis

We used Statistical Analysis System software v9.4 (SAS Institute Inc., Cary, NC) for data analysis. Qualitative variables were quoted as absolute numbers and relative frequencies. To test the association between potential perceived differences in harm caused by e-cigarettes and tobacco cigarettes and possible associations between education and perceptions of e-cigarette harm, we conducted the likelihood ratio chi-square and Fisher exact test using 2*2 and r*c contingency tables. To test the difference between the two groups, we used a Z-test, with null hypothesis being no difference. Bonferroni or false discovery rate approaches were applied to counteract the multiple comparisons problem.

We used generalized linear models with appropriate link functions to test the association between outcome and predictor variables. All statistical tests comparing two groups were conducted using a two-tailed approach. Test results were deemed statistically significant if *P* ≤ .05. Predictor variables significant at the bivariate level were entered into the multivariable model.

### Missing Data

One survey from the medical student group had missing data. This was assumed to be random and excluded after a complete-case analysis.

## RESULTS

### Demographics and Use History

Ninety surveys were returned from the ED patient population. A total of 14,429 patients between the ages of 18–35 were seen in the ED during the survey dates. Data for vaping status for all patients was not available. Under the assumption that 15% of patients in this age range, or ~2,164 individuals, would be eligible for the study, the survey achieved a response rate of approximately 4.2% for the ED population.^15^ Of the 1,055 medical students who were administered the survey, 187 surveys were returned, with a response rate of 17.7%. Table 1 contains demographic information for ED patients and medical students.

Medical students were younger than the ED patients queried (Table 1). Post-hoc analysis of individual age groups revealed significant differences across all age groupings. Patients in the ED were more likely to identify as non-binary/prefer not to answer (8.9%, n = 8 vs 4.0%, n = 11, *P* = .004). There was a significant difference in race distribution, with more Black respondents in the ED patient group (24.4%, n = 22 vs 8.7%, n = 24, *P* < .001), while White and Asian respondents were more represented in the medical student group (*P* = .002 for both, Table 1). The highest level of education was significantly different between the two populations. Of the 43% of medical students who reported e-cigarette use, most reported using e-cigarettes for six months or less, while most of the ED patients used e-cigarettes for one year or longer. Additionally, a larger proportion of ED patients than medical students identified frequent (daily) e-cigarette use (31.1% vs 11.9%, *P* < .001).

### Aim 1: Perceptions of health effects among young ED patients who use e-cigarettes and comparing perceptions between ED patients and medical students

A moderate proportion of ED patients reported their belief that e-cigarettes were not capable of causing lung injury (22.2%), heart disease (30.0%), or cancer (17.8%) (Table 2). When evaluating perceived harm relative to cigarettes, ED respondents perceived e-cigarettes as similarly capable of causing harm with 41.1% saying e-cigarettes are equally as likely to cause lung injury, 56.7% saying e-cigarettes are equally as likely to cause heart disease, and 55.6% saying e-cigarettes are equally as likely to cause cancer.

Compared to all medical students, fewer ED patients perceived e-cigarettes as capable of causing lung injury, heart disease, and cancer. When compared to the subgroup of medical students who had tried e-cigarettes (n = 81), the difference in perception of lung injury persisted (77.8% vs 96.3%; *P* < .001), but there was no significant difference in perceptions of e-cigarettes’ ability to cause cancer (82.2% vs 84.0%; *P* = .63) and heart disease ((70.0% vs 79.0%, *P* = .18).

In evaluating perceived harm relative to cigarettes, ED patients were significantly more likely than all medical students to report e-cigarettes as more dangerous than cigarettes in terms of lung injury (35.6% vs 23.8%, *P* = .001), heart disease (22.2% vs 12.6%, *P* = .001), and cancer (15.6% vs 9.8%, *P* = .02) (Table 3). And when comparing to the portion of medical students with e-cigarette use history, still more ED patients perceived e-cigarettes as more capable than cigarettes of causing lung injury (35.6% vs 12.3%, *P* < .001), heart disease (22.2% vs 7.4%, *P* = .007), and cancer (15.6% vs 2.5%, *P* = .002).

Twelve of the 16 medical students (75%) who did not perceive e-cigarettes to be capable of causing cancer had tried e-cigarettes. Of the 169 medical students (90.4%) who believed e-cigarettes could increase risk of cancer, 68 (40%) had tried an e-cigarette before. The 173 students who perceived e-cigarettes as more likely than cigarettes to cause cancer were less likely to have tried e-cigarettes, as only 45.7% (*P* = .025) of them had ever used e-cigarettes; no significant associations were found between having tried an e-cigarette and perceptions of e-cigarettes compared to cigarettes’ ability to cause lung injury or heart disease.

### Aim 2: Medical students’ views on curriculum and counseling confidence

More than half of students (54.0%) expressed low confidence in advising patients about the potential dangers of e-cigarette use, and 36.9% of students were not confident in counseling on nicotine product use in general. Sixty-one percent of medical students felt that the medical school curriculum did not adequately address e-cigarette topics. When broken down by phase in school (pre-clinical vs clinical), there was no significant difference in confidence with counseling; however, there was a significant difference in perception of the curriculum with a larger proportion of clinical students (77.1%) finding the curriculum inadequate compared to the pre-clinical students (57.2%, *P* = .001). When comparing students who had tried an e-cigarette to those who had not, there was no significant difference in counseling confidence or opinion of the medical school curriculum.

## DISCUSSION

### Aim 1: Perceptions of health effects among young ED patients who used e-cigarettes compared to medical students and implications for development of brief intervention

A significant proportion of ED patients reported that e-cigarettes would not cause lung injury (22%), heart disease (30%), or cancer (18%). Emergency department patients in this cohort also perceived e-cigarettes as equally or less harmful than traditional cigarettes. Other research has demonstrated that adolescents and younger adults hold perceptions that e-cigarettes tend to have less associated health risk and less addictive potential as compared to traditional cigarettes, increasing the prevalence of initiation, adherence, and future use.^16^–^18^ Additionally, multiple studies reported similar proportions of younger adults perceiving e-cigarettes as less harmful than and equally as harmful as cigarettes.^19^, ^20^

Previous data have indicated that awareness of potential health effects leads to reduced use.^21^ While long-term health effects data are limited, reports have demonstrated that an increased risk of developing adverse health conditions exists with e-cigarette use. Thus, knowledge gaps about potential health risks have become the key barrier to reducing e-cigarette use, illustrating the importance of accessible information to educate e-cigarette users or at-risk populations.

Our data suggest that medical education may increase awareness of health risks associated with e-cigarettes, with medical students being more likely to perceive risk from their use, although both cohorts perceived e-cigarettes as less harmful than tobacco cigarettes. This finding is consistent with prior research, such as a study conducted at the University of Arkansas for Medical Sciences, where a significant portion of students in health degree programs also believed e-cigarettes were less harmful than cigarettes.^26^ This perception may reflect broader public narratives that frame e-cigarettes as a “safe” alternative, even though emerging evidence points to serious health risks.

In our cohort, medical students who recognized the potential harms of e-cigarettes were significantly less likely to have ever tried them. This supports the idea that greater awareness of the health risks is associated with lower usage rates, reinforcing the idea that improving public knowledge could be a powerful tool in curbing e-cigarette use. Emergency department patients who attained a lower level of education reported lower awareness of the potential health risks of e-cigarettes. Whether this is specifically secondary to education level is unclear, but it supports previous research that those with lower education levels are more likely to use e-cigarettes.^2^ Targeted education campaigns, as well as ED-based initiatives aimed at increasing knowledge about both the immediate and long-term risks of e-cigarette use, could help shift perceptions and ultimately reduce use.

Data gathered in this study may help influence the development of targeted brief interventions for ED patients who use e-cigarettes. Multiple studies have shown that ED interventions are successful in identifying and curbing substance use rates for traditional cigarettes, marijuana, and alcohol. For example structured screening tools, such as SBIRT (screening, brief intervention, and referral), have been successful in effectively detecting high-risk substance use while simultaneously reducing risky behaviors and motivating treatment engagement.^22^ Regarding e-cigarette use, consensus-based clinical guidance for e-cigarette cessation has been established.^23^ Incorporating these recommendations, as well as validated screening tools, allows the ED to offer a solution to initiate e-cigarette cessation efforts effectively.

Previously established programs, such as the UKanQuit program at the University of Kansas Medical Center, provide tobacco cessation services to hospitalized patients, offering bedside counseling, nicotine replacement, and medication management.^24^ A 2010 observational study evaluating the program reported that 31.8% of participants achieved 7-day point prevalence abstinence at 6-month follow-up, decreased the average number of cigarettes smoked per day significantly from 17.8 to 14.0, and revealed that 70% of participants made at least one quit attempt lasting 24 hours or more post-discharge.^25^ This study group aims to use the data generated in this study to develop and implement an ED-based brief intervention, adhering to similar services offered by the UKanQuit program. The intervention will target the surveyed health effects to educate e-cigarette users presenting in the ED, as their perceptions of harm from e-cigarette use are limited.

### Aim 2: Medical students’ views on curriculum and counseling confidence

Our findings suggest that medical students are generally aware of the potential health effects associated with e-cigarette use, with an average of 90% of students reporting e-cigarettes as capable of causing the surveyed health effects; however, their confidence in advising patients about possible health risks was low. This gap may lie in the medical school curriculum, as many students felt their education did not sufficiently address e-cigarette use and potential hazards. Currently, the students have a total of four hours within the preclinical curriculum dedicated to e-cigarette related topics.

Despite growing recognition of the potential for harmful effects of e-cigarettes within the scientific community, medical education on the topic remains limited. In a pilot survey of 259 medical students, 68.7% rated their e-cigarette-related medical education as “inadequate,” and 76.1% reported that their curriculum had no impact on their views or behaviors around e-cigarette use.^27^ Similarly, in a needs analysis of 31 healthcare professionals, non-experts and experts alike reportedly felt they required additional training in clinical skills for addressing patient e-cigarette use.^28^ Echoing Ruppel et al’s observation that e-cigarette education is largely insufficient, our study likewise found the current curriculum to be inadequate. Given these findings, medical education should adapt so that professionals can address the growing public health concerns surrounding e-cigarette use.[Table t4-wjem-27-564]

Integrating more detailed and up-to-date information on the potential health risks of e-cigarette use into the curriculum could significantly improve students’ confidence in counseling patients and, ultimately, contribute to better patient education and outcomes. In addition, the curriculum should aim to teach students how to counsel patients when limited data are available. Future physicians must be equipped with the knowledge and skills to provide practical guidance, especially as e-cigarette use rises. Addressing these educational gaps will better prepare future physicians to take an active role in preventing e-cigarette-related illnesses and advocating for public health.

## LIMITATIONS

Key limitations to this study are the sample size and the populations surveyed. Because the ED patient sample was small, analyses were limited. Limited response rates may be attributable to challenges in implementation and inconsistent incorporation of the protocol into usual workflows. In the same fashion, sicker patients may have been missed due to emergent prioritization. Due to the small sample of ED patients, the data may not represent a greater population and should be viewed as exploratory. Results from this cohort may not be similar across similar aged cohorts in other parts of the U.S.; therefore, we suggest a larger effort to explore health risk perceptions to determine optimal areas for intervention. In addition, the response rate was calculated based on an estimate of patients who may have been eligible for the study, as the number of patients approached to participate is not available. This may introduce non-response bias into the sample, which we are unable to account for.

Emergency departments tend to have a patient population that is less educated and has a lower overall socioeconomic status, which may or may not be representative of the general e-cigarette user. However, these patients are a population for which targeted education may improve health outcomes. Along with the disparities of racial and ethnic diversity in medical school education, the likelihood of lower socioeconomic status among the ED patient sample leads to decreased generalizability. In addition, patients were recruited from a single ED in a major metropolitan area, and medical students were recruited from a single medical school. There were also racial and age differences between medical students and ED patients, which were not further investigated within the parameters of this study.

This study did not directly assess whether participants were current users or, if they had quit using e-cigarettes, why they stopped. Understanding what led to cessation and potential changes in perceptions of e-cigarettes during an individual’s time using them could be influential in education initiatives. Finally, survey methodology creates the potential for recall bias and possible under/over-reporting of use.

## CONCLUSION

This study explores perceptions of e-cigarette use in young ED patients and underscores differences in perceptions and usage between medical students and ED patients. A modest portion of ED patients did not perceive e-cigarettes as capable of causing adverse health outcomes and reported that e-cigarettes could be a safe alternative to cigarettes, whereas medical students perceived greater potential harm from e-cigarette use. Understanding the demographic and educational factors driving these disparities can guide the development of targeted policies and interventions moving forward. Most of the medical students who completed the survey reported low confidence in counseling patients about e-cigarette use, indicating that current medical curricula inadequately address the hazards of smoking e-cigarettes. While limitations of sample size and demographic difference may limit generalizability of these findings, these insights may nevertheless help inform the design of an ED-based brief intervention aimed at promoting e-cigarette cessation as well as improvements in medical education regarding health risks associated with e-cigarette use.

## Figures and Tables

**Table 1:** Participant demographics, separated by surveyed group (patients vs. medical students), in a study of perceived health risks associated with e-cigarette use.

	Percentage (Frequency)			
Age in years	All respondents	ED patients	Medical students	*P* value
18–23	40.1% (111)	33.3% (30)	43.3% (81)	.11
24–29	45.1% (125)	34.4% (31)	50.3% (94)	.013
30–35	14.4% (40)	31.1% (28)	6.4% (12)	< .001
Missing	0.4% (1)	1.1% (1)		
Sex				
Female	58.1% (161)	52.2% (47)	61.0% (114)	.17
Male	37.5% (105)	38.9% (35)	37.4% (70)	.81
Non-binary/prefer not to answer	4.0% (11)	8.9% (8)	1.6% (3)	.004
Race				
White	73.3% (203)	61.1% (55)	79.1% (148)	.002
American Indian/Alaska Native	2.1% (6)	2.2% (2)	2.1% (4)	.96
Black	8.7% (24)	24.4% (22)	1.1% (2)	< .001
Asian	6.9% (19)	0.0% (0)	10.2% (19)	.002
Native Hawaiian or Pacific Islander	0.3% (1)	1.1% (1)	0.0% (0)	.15
More than one race	8.7% (24)	11.1% (10)	7.5% (14)	.32
Ethnicity				
Not Hispanic or Latino	80.9% (224)	76.7% (69)	82.9% (155)	.22
Hispanic or Latino	15.5% (43)	20.0% (18)	13.4% (25)	.16
Middle Eastern/North African	3.6% (10)	3.3% (3)	3.7% (7)	.87
Highest level of education				
Complete high school degree or less	16.2% (45)	50.0% (45)	0.0% (0)	< .001
Some college	7.9% % (22)	24.4% (22)	0.0% (0)	< .001
Completed post-secondary degree or higher	75.8% (210)	25.6% (23)	100.0% (187)	.001
Ever used an e-cigarette				
Yes	62.0% (171)	100.0% (90)	43.5% (81)	< .001
No	38.0% (105)	0% (0)	56.5% (105)	< .001
Missing	0.4% (1)	0% (0)	0.5% (1)	
Length of time of e-cigarette use				
Never used	40.2% (111)	0.0% (0)	59.7% (111)	< .001
0–6 months	23.6% (65)	27.8% (25)	21.5% (40)	.24
6–12 months	5.4% (15)	10.0% (9)	3.2% (6)	.02
1–2 years	9.1% (25)	18.9% (17)	4.3% (8)	< .001
3–5 years	10.1% (28)	20.0% (18)	5.4% (10)	< .001
≥ 5 years	9.4% (26)	15.6% (14)	6.5% (12)	.01
Missing	2.5% (7)	7.8% (7)	0% (0)	
Frequency of e-cigarette use				
Daily or almost daily	12.0% (33)	31.1% (28)	2.7% (5)	<.001
Less than daily, but at least once a week	3.6% (10)	10.0% (9)	0.5% (1)	<.001
Less than weekly, but at least once a month	2.2% (6)	2.2% (2)	2.1% (4)	.96
Less than monthly	8.0% (22)	8.9% (8)	7.5% (14)	.69
Not at all	74.6% (206)	47.8% (43)	87.6% (163)	< 001

*ED*, emergency department.

**Table 2:** Perceptions of health effects from e-cigarette use among young adult emergency department patients and medical students in a study of perceptions of the health risk of e-cigarette use.

	Percentage (Frequency)			
E-cigarette use can cause lung injury	All respondents	ED patients	Medical students	*P* value
Yes	89.2% (247)	77.8% (70)	94.7% (177)	< .001
No/not sure	10.8% (30)	22.2% (20)	5.3% (10)	< .001
E-cigarette use can cause heart disease				
Yes	79.4% (220)	70.0% (63)	84.0% (157)	.007
No/not sure	20.6% (57)	30.0% (27)	16.0% (30)	.007
E-cigarette use can cause cancer				
Yes	88.1% (244)	82.2% (74)	90.9% (170)	.04
No/not sure	11.6% (32)	17.8% (16)	8.6% (16)	.03
Missing	0.4% (1)	0% (0)	0.5% (1)	

*ED*, emergency department.

**Table 3:** Perceptions of health risks associated with e-cigarette vs. traditional cigarette use in a study of health risk perceptions associated with e-cigarette use among young adult emergency department patients and medical students.

	Percentage (Frequency)			
E-cigarettes’ health effects vs cigarettes	All respondents	ED patients	All medical students	*P* value
E-cigarettes are healthier than cigarettes	35.7% (99)	35.5% (32)	35.8% (67)	.96
E-cigarettes are about the same as cigarettes	42.6% (118)	31.1% (28)	48.1% (90)	.007
E-cigarettes are more harmful than cigarettes	21.7% (60)	33.3% (30)	16.0% (30)	.001
Likelihood to cause lung injury				
E-cigarettes are more likely than cigarettes	23.8% (66)	35.6% (32)	18.2% (34)	.001
E-cigarettes are about the same as cigarettes	46.9% (130)	41.1% (37)	49.7% (93)	.07
E-cigarettes are less likely than cigarettes	29.3% (81)	23.4% (21)	32.1% (60)	.18
Likelihood to cause heart disease				
E-cigarettes are more likely than cigarettes	12.6% (35)	22.2% (20)	8.0% (15)	.001
E-cigarettes are about the same as cigarettes	50.2% (139)	56.7% (51)	47.1% (88)	.13
E-cigarettes are less likely than cigarettes	37.2% (103)	21.1% (19)	44.9% (84)	< .001
Likelihood to cause cancer				
E-cigarettes are more likely than cigarettes	9.8% (27)	15.6% (14)	7.0% (13)	.02
E-cigarettes are about the same as cigarettes	42.2% (117)	55.6% (50)	35.8% (67)	.002
E-cigarettes are less likely than cigarettes	48.0% (133)	28.8% (26)	57.2% (107)	< .001

*ED*, emergency department.

**Table 4 t4-wjem-27-564:** Perceptions of medical students toward medical school curriculum and preparedness to counsel patients on nicotine and e-cigarette use.

	Percentage (Frequency)
Believe medical school curriculum inadequately covers e-cigarette topics	61.0% (114)
Not confident counseling on nicotine use	36.9% (69)
Not confident counseling on e-cigarette use	54.0% (101)
